# Cell-Penetrating Delivery of Nitric Oxide by Biocompatible Dinitrosyl Iron Complex and Its Dermato-Physiological Implications

**DOI:** 10.3390/ijms221810101

**Published:** 2021-09-18

**Authors:** Yu-Chieh Chen, Yi-Hong Chen, Han Chiu, Yi-Hsuan Ko, Ruei-Ting Wang, Wei-Ping Wang, Yung-Jen Chuang, Chieh-Cheng Huang, Tsai-Te Lu

**Affiliations:** 1 Department of Medical Science & Institute of Bioinformatics and Structural Biology, National Tsing Hua University, Hsinchu 30013, Taiwan; jeremy80808@gmail.com (Y.-C.C.); 0953665406irisko@gmail.com (Y.-H.K.); yjchuang@life.nthu.edu.tw (Y.-J.C.); 2 Institute of Biomedical Engineering, National Tsing Hua University, Hsinchu 30013, Taiwan; chenyh@gapp.nthu.edu.tw (Y.-H.C.); chiuhan911@gmail.com (H.C.); 3 CHLITINA Research and Development Center, CHLITINA Holding Ltd., Taipei 10073, Taiwan; mia.wang@tw.chlitina.com (R.-T.W.); wpw.wang@tw.chlitina.com (W.-P.W.)

**Keywords:** nitric oxide, dinitrosyl iron complex, controlled delivery, wound healing, collagen deposition

## Abstract

After the discovery of endogenous dinitrosyl iron complexes (DNICs) as a potential biological equivalent of nitric oxide (NO), bioinorganic engineering of [Fe(NO)_2_] unit has emerged to develop biomimetic DNICs [(NO)_2_Fe(L)_2_] as a chemical biology tool for controlled delivery of NO. For example, water-soluble DNIC [Fe_2_(μ-SCH_2_CH_2_OH)_2_(NO)_4_] (**DNIC-1**) was explored for oral delivery of NO to the brain and for the activation of hippocampal neurogenesis. However, the kinetics and mechanism for cellular uptake and intracellular release of NO, as well as the biocompatibility of synthetic DNICs, remain elusive. Prompted by the potential application of NO to dermato-physiological regulations, in this study, cellular uptake and intracellular delivery of DNIC [Fe_2_(μ-SCH_2_CH_2_COOH)_2_(NO)_4_] (**DNIC-2**) and its regulatory effect/biocompatibility toward epidermal cells were investigated. Upon the treatment of **DNIC-2** to human fibroblast cells, cellular uptake of **DNIC-2** followed by transformation into protein-bound DNICs occur to trigger the intracellular release of NO with a half-life of 1.8 ± 0.2 h. As opposed to the burst release of extracellular NO from diethylamine NONOate (DEANO), the cell-penetrating nature of **DNIC-2** rationalizes its overwhelming efficacy for intracellular delivery of NO. Moreover, NO-delivery **DNIC-2** can regulate cell proliferation, accelerate wound healing, and enhance the deposition of collagen in human fibroblast cells. Based on the in vitro and in vivo biocompatibility evaluation, biocompatible **DNIC-2** holds the potential to be a novel active ingredient for skincare products.

## 1. Introduction

Based on the distinctive electron paramagnetic resonance (EPR) signal at g ≈ 2.03 observed in the baker’s yeast cells (*S. cerevisiae*), biosynthesis of dinitrosyl iron complexes (DNICs) was first discovered by Prof. Vanin in 1964 [1,2,3,4]. Later, continued investigations explored tetrahedral DNICs [(NO)_2_Fe(L)_2_] as a natural and ubiquitous cofactor generated upon the interaction of nitric oxide (NO) with non-heme [Fe-S] proteins and cellular labile iron pools [5,6,7,8,9,10,11,12,13,14,15,16,17,18]. Despite the identification of NO as an endothelium-derived relaxing factor (EDRF) [19,20], DNICs in a low-molecular-weight or protein-bound form were proposed as a “working form” of NO under physiological conditions [21,22]. As a potential biological equivalent of NO, bioinorganic engineering of [Fe(NO)_2_] unit has emerged to develop biomimetic DNICs as a chemical biology tool for controlled delivery of NO and translation of NO-related functions into biomedical applications [23,24]. In particular, biomimetic DNICs and their conjugates with drug delivery systems were explored for anti-aging/anti-inflammatory/anti-viral effect, anti-cancer/anti-hypertensive therapy, diabetic angiogenesis/wound healing, penile erection, and treatment of mild cognitive impairment and neurodegenerative diseases [25,26,27,28,29,30,31,32,33,34,35]. In particular, phase I and phase II clinical trials of glutathione-bound DNICs [Fe_2_(μ-glutathione)_2_(NO)_4_], a commercial medical product with the pharmacological name Oxacom^®^, was reported for application as a clinical medicine against hypertension [30,31]. 

Inspired by the potential of biomimetic DNICs for biomedical applications, synthetic advances in the development of structure-characterized and water-soluble DNICs enabled the study of trafficking and NO-delivery reactivity of DNICs under simulated physiological conditions, in vitro, and in vivo [26,28,36,37,38,39]. Water-soluble DNIC [Fe_2_(μ-SCH_2_CH_2_OH)_2_(NO)_4_] (**DNIC-1**) displays an O_2_-triggered degradation mechanism for the release of nitric oxide, ferric ion, and disulfide with a half-life of 27.4 h and 15.5 h in an aqueous buffer solution (pH = 7.0 or 7.4) at 25 °C and 37 °C, respectively [25,32,34]. In simulated gastric fluid (SGFsp, pH 1.2), a shortened half-life of 0.4 h indicates the acid-sensitive nature of **DNIC-1**. To mimic the systemic circulation system, the addition of serum albumin initiated the coordination of Cys-34 toward **DNIC-1** leading to the partial formation of albumin-bound DNIC, which features an accelerated release of NO with a half-life of 3.5 h in phosphate-buffered saline (PBS) [34,39,40,41,42]. Regarding the NO-release reactivity of synthetic DNICs, their capability for the elevation of intracellular NO levels, activation of the NO-soluble guanylyl cyclase (sGC)-cyclic guanosine monophosphate (cGMP) pathway (NO-sGC-cGMP pathway), and regulation of cGMP-dependent cell proliferation was also reported [32,34,39,43]. Moreover, the discovery of multidrug-resistance-associated protein 1 as a transporter for the efflux of glutathione-bound DNICs from the cells may establish a mechanism for transcellular trafficking of DNIC after its uptake by cells [44,45,46]. Of importance, the endogenous conjugation of **DNIC-1** with gastrointestinal mucin and serum albumin in a reversible manner was reported to facilitate oral delivery of the [Fe(NO)_2_] unit to the brain across the intestinal epithelial and blood-brain barriers, respectively [34]. Despite the investigations put forward, kinetics for in vitro NO-release reactivity, the mechanism for cellular uptake and intracellular release of NO, and the biocompatibility of synthetic DNICs remain elusive.

**Chart 1:**
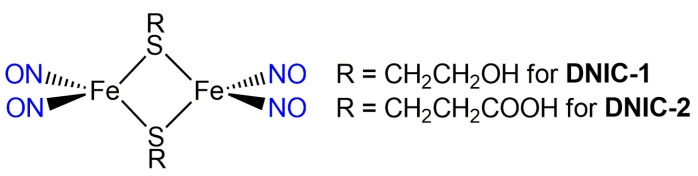
Structure of DNICs [Fe_2_(μ-SCH_2_CH_2_OH)_2_(NO)_4_] (**DNIC-1**) and [Fe_2_(μ-SCH_2_CH_2_COOH)_2_(NO)_4_] (**DNIC-2**).

Prompted by the potential application of NO to dermato-physiological regulations [47,48,49,50], herein, the mechanism for intracellular delivery of DNIC [Fe_2_(μ-SCH_2_CH_2_COOH)_2_(NO)_4_] (**DNIC-2**) and its regulatory effect/biocompatibility toward epidermal cells were investigated (Chart 1). Upon the treatment of **DNIC-2** to human fibroblast cells, cellular uptake of **DNIC-2** and transformation into protein-bound DNICs occur to trigger the intracellular release of NO with a half-life of 1.8 ± 0.2 h. As opposed to the burst release of extracellular NO from diethylamine NONOate (DEANO), the cell-penetrating nature of **DNIC-2** rationalizes its overwhelming efficacy for intracellular delivery of NO. Despite the absent regulation of pigmentation of B16-F10 melanoma cells, notable effects on cell proliferation, accelerated wound healing, and the enhanced deposition of collagen were observed upon treatment of **DNIC-2** to human fibroblast cells. Based on the biocompatibility evaluation using a 2-D culture of fibroblast/melanocyte/keratinocyte cells, 3-D culture of reconstructed human epidermis and human cornea-like epithelium models, and zebrafish embryo, **DNIC-2** holds the potential for further development as a novel active ingredient for skincare products. 

## 2. Results and Discussion 

### 2.1. Cellular Uptake of Dinitrosyl Iron Complex (DNIC) [Fe_2_(μ-SCH_2_CH_2_COOH)_2_(NO)_4_] (DNIC-2) and Intracellular Release of Nitric Oxide (NO)

#### 2.1.1. Kinetic Study of NO-Release Reactivity for DNIC-2

**Figure 1:**
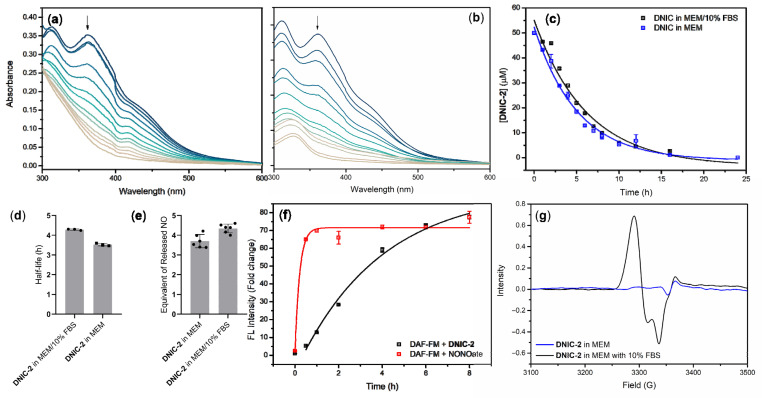
Kinetics and efficacy study of NO-release reactivity of [Fe_2_(μ-SCH_2_CH_2_COOH)_2_(NO)_4_] (**DNIC-2**). Time-dependent change of UV-vis spectra for **DNIC-2** in MEM (**a**) with and (**b**) without the presence of 10% FBS. The arrows indicate the time-dependent change of the UV-vis spectra. (**c**) Decompositions of **DNIC-2** in MEM with (black) and without (blue) the presence of 10% FBS, respectively, which were fitted to pseudo-first-order kinetics. Data show the mean ± SD from three independent experiments. (**d**) Half-life for decomposition of **DNIC-2** in MEM with or without the presence of 10% FBS at 37 °C. Data represent the mean ± SD (*n* = 3). (**e**) Equivalent of released nitric oxide during degradation of **DNIC-2**. The data are the mean values ± SD pooled from six independent experiments. (**f**) Time-dependent change of relative fluorescence intensity of NO-specific probe DAF-FM (excitation at 495 nm and emission at 535 nm) with the treatment of 50 μM of **DNIC-2** (black) or 133 μM of NONOate (red) in MEM with 10% FBS, which were fitted to pseudo-first-order kinetics. Data show the mean ± SD from three independent experiments. (**g**) EPR spectra for **DNIC-2** in MEM with (black) and without (blue) the presence of 10% FBS, respectively.

Kinetics and efficacy for the release of NO from **DNIC-2** in a cell culturing medium, the minimum essential medium (MEM), were investigated to project on the in vitro study discussed below. Similar to the reported DNIC [Fe_2_(μ-SCH_2_CH_2_OH)_2_(NO)_4_] (**DNIC-1**) [25,34], **DNIC-2** exhibits a steady and aerobic decomposition with a half-life of 4.2 ± 0.6/3.5 ± 0.3 h in MEM with/without the presence of 10% fetal bovine serum (FBS) (Figure 1a–d). Moreover, this aerobic degradation of **DNIC-2** results in a concomitant release of ~4 equiv. of NO based on the total nitrite/nitrate assay (Figure 1e). In addition, the aerobic decomposition of **DNIC-2** and release of NO was also validated using an NO-specific fluorescence probe, 4-amino-5-methylamino-2′,7′-difluorofluorescein diacetate (DAF-FM), whereas the NO donor diethylamine NONOate (DEANO) was used for comparison. Regarding the stoichiometry of released NO (≈4 equiv. for **DNIC-2** and 1.5 equiv. for DEANO), reactions of DAF-FM with 50 μM of **DNIC-2** and 133 μM of DEANO, respectively, were further performed. In comparison with DAF-FM only, both **DNIC-2** and DEANO trigger a time-dependent and significant increase of fluorescence intensity (Figure 1f). Relevant to the reported **DNIC-1** [34], presumably, O_2_-induced oxidation of the [Fe(μ-SR)_2_Fe] core in **DNIC-2** weakens the Fe-to-NO π-backbonding interaction and initiates the release of NO as probed by DAF-FM under aerobic conditions [51]. In the absence of an NO-responsive target (i.e., DAF-FM), the subsequent oxidation of released NO results in the formation of nitrite/nitrate.

With regard to the reported interconversion between low-molecular-weight and protein-bound DNICs [7,34,52,53], the formation of protein-bound DNIC derived from **DNIC-2** in MEM with/without the presence of 10% FBS was further explored. As opposed to the EPR silence displayed by **DNIC-2** in MEM, the addition of 10% FBS results in the assembly of protein-bound DNIC based on the formation of a rhombic EPR signal at g = 2.043, 2.036, and 2.015 (Figure 1g). Using DNIC [PPN][(NO)_2_Fe(S_5_)] (PPN = bis(triphenylphosphine)iminium, S_5_ = pentasulfide) as a standard, spin quantitation of this protein-bound DNIC determines a 1.5%-conversion of low-molecular-weight **DNIC-2** into the protein-bound form (Figure S1). In comparison with other protein-bound and biomimetic DNICs [(NO)_2_Fe(SR)(L)]^−^ [54], serum albumin is proposed to facilitate this minor formation of protein-bound DNIC upon incubation of **DNIC-2** in MEM with the presence of 10% FBS, in which the concentration of serum albumin is 2.1 mg/mL (Tables S1 and S2). This minor formation of protein-bound DNIC, moreover, rationalizes the comparable half-life and efficacy for the release of NO from **DNIC-2** in MEM with or without the presence of 10% FBS discussed above. Considering 10% FBS as an essential ingredient, the in vitro study described in the following sections was performed in MEM with the presence of 10% FBS. 

#### 2.1.2. Cellular Uptake of DNIC-2 and Intracellular Release of NO in CCD-966SK Human Skin Fibroblast Cells

**Figure 2:**
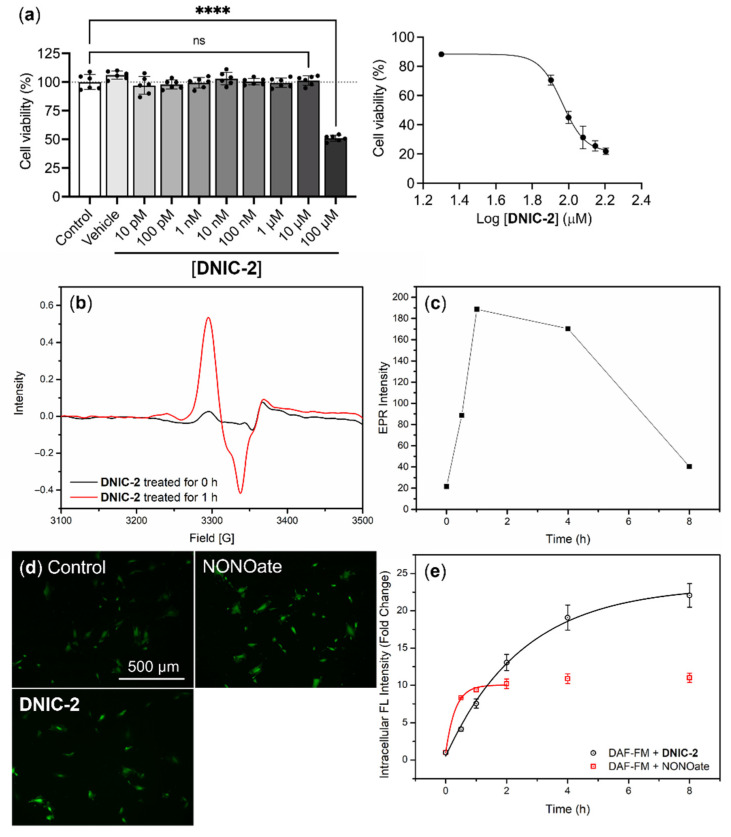
Cellular uptake of **DNIC-2** and intracellular release of nitric oxide in human skin fibroblast (CCD-966SK) cells. (**a**) Cell viability assay of the human skin fibroblast (CCD-966SK) cells treated with different concentrations of **DNIC-2** for 24 h **** *p* < 0.001 compared to the group without treatment of **DNIC-2**. (**b**) Representative EPR spectra for CCD-966SK cells treated with 50 μM of **DNIC-2** for 0 h (black) and 1 h (red). (**c**) Formation and decay of protein-bound DNIC in CCD-966SK cells after treatment of 50 μM of **DNIC-2** for 0, 0.5, 1, 4, and 8 h. (**d**) Fluorescence images of CCD-966SK cells treated with DAF-FM (10 μM) for 1 h, with DAF-FM (10 μM) and **DNIC-2** (50 μM) for 4 h, and with DAF-FM (10 μM) and NONOate (133 μM) for 1 h, respectively. (**e**) Time-dependent change of fluorescence intensity of CCD-966SK cells treated with 10 μM of DAF-FM and 50 μM of **DNIC-2** (black) or with 10 μM of DAF-FM and 133 μM of NONOate (red) for 0, 0.5, 1, 2, 4, and 8 h, which were fitted to pseudo-first-order kinetics. Data show the mean ± SD from three independent experiments.

As shown in Figure 2a, **DNIC-2** exhibits an IC_50_ value of 92.3 μM toward CCD-966SK human skin fibroblast cells based on the CCK-8 assay. Accordingly, 50 μM of **DNIC-2** was further treated to CCD-966SK cells in the attempt to explore the cellular uptake of **DNIC-2** followed by the intracellular release of NO. Upon the addition of 50 μM of **DNIC-2** to CCD-966SK cells, the time-dependent formation of EPR signal at g = 2.041, 2.034, and 2.015 indicates the cellular uptake of **DNIC-2** followed by intracellular transformation of **DNIC-2** into protein-bound DNICs, of which the level starts to decay after treatment of **DNIC-2** for 1 h (Figure 2b,c). Through the sequential incubation of CCD-966SK cells with an NO-specific probe DAF-FM and **DNIC-2**, the elevated generation of intracellular fluorescence signal demonstrates the successful delivery of NO into CCD-966SK cells by **DNIC-2** (Figure 2d). Moreover, a 22-fold increase of intracellular fluorescence intensity in a steady manner reflects that **DNIC-2** features a half-life of 1.8 ± 0.2 h for cell-penetrating delivery of NO in CCD-966SK cells (Figure 2e). This contrasts with the instantaneous formation of a maximum 10-fold increase of fluorescence intensity observed after treatment of DEANO, which features a half-life of 0.2 ± 0.1 h (Figure 2d,e). That is, **DNIC-2** features a steady and more effective cell-penetrating delivery of NO overwhelming the DEANO. Presumably, cellular uptake of **DNIC-2** and its transformation into protein-bound DNICs followed by the intracellular release of NO may rationalize its overwhelming efficacy for the cell-penetrating delivery of NO. Considering that the biological targets responsive for NO-related physiology (i.e., soluble guanylyl cyclase (sGC)) are located in the intracellular compartment, the potential of effective NO-delivery **DNIC-2** prompts our in vitro investigations on its dermato-physiological applications. 

### 2.2. NO-Delivery DNIC-2 Promoted the Proliferation, Migration and Collagen Deposition of Skin Fibroblasts

**Figure 3:**
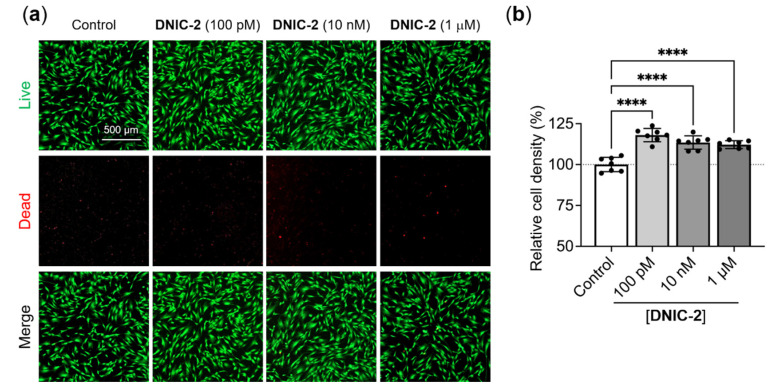
Low-dose **DNIC-2** promotes the proliferation of human skin fibroblasts. (**a**) Representative live/dead staining images of **DNIC-2**-treated CCD-966SK cells and (**b**) their corresponding cell densities determined by counting the number of DAPI stained nuclei (*n* = 7). The data are presented as mean ± SD. **** *p* < 0.001.

To evaluate the potential of the developed NO donor for dermatological application, we first investigated the effects of low-dose **DNIC-2** on the behaviors of CCD-966SK cells. After a 48-h incubation, a higher cell density was observed in the groups that received the treatment of **DNIC-2** than that of the untreated control (Figure 3a). Furthermore, the fibroblasts that were treated with 100 pM, 10 nM, and 1 μM **DNIC-2** showed 18.1 ± 4.1%, 13.5 ± 4.0%, and 12.2 ± 2.4% increases in cell density, respectively (*p* < 0.001; Figure 3b), suggesting that **DNIC-2** could efficiently promote fibroblast proliferation. Although the effects of NO on modulating skin fibroblast proliferation were reportedly controversial [47,48,49,55,56], most studies employed short-term NO donors that evolve NO for only several minutes. In the present study, however, the developed **DNIC-2** with a half-life of ~4 h for the steady release of NO, thus, features a distinct impact on fibroblast proliferation.

**Figure 4:**
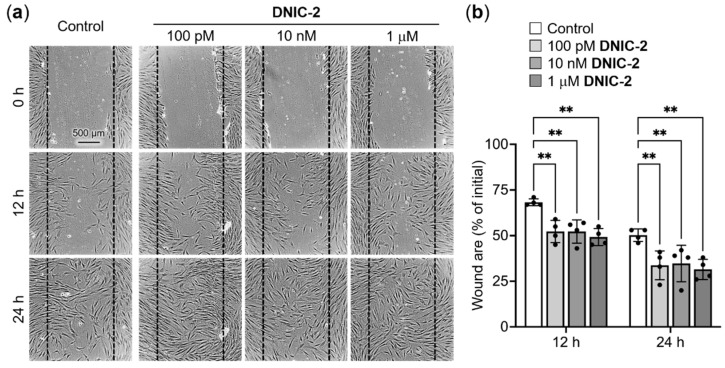
Low-dose **DNIC-2** accelerates the migration of human skin fibroblasts. (**a**) Representative phase-contrast photomicrographs of **DNIC-2**-treated CCD-966SK cells in a scratch wound healing assay and (**b**) their corresponding wound area (*n* = 4). The data are presented as mean ± SD. ** *p* < 0.05.

The potential of exogenously administered NO in accelerating fibroblast migration, a key step involved in skin wound healing [57], has been well-documented [50,55]. Herein, an in vitro scratch wound healing assay was conducted to analyze the capacity of **DNIC-2** in promoting fibroblast migration. After creating a wound gap on a confluent culture using a sterilized P200 tip, the CCD-966SK cells were treated with the media that contained **DNIC-2** before being incubated for 24 h. As revealed in Figure 4a,b, more cells were observed in the gap area in **DNIC-2**-treated groups than that of the untreated control (*p* < 0.05), indicating the capacity of **DNIC-2** in promoting fibroblast migration.

**Figure 5:**
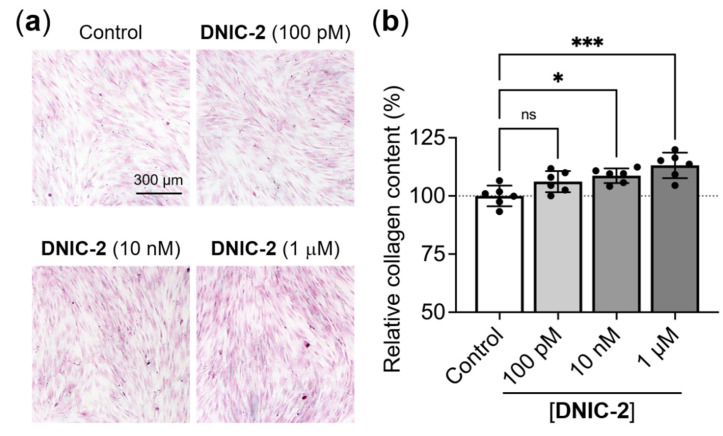
Low-dose **DNIC-2** enhances the collagen deposition of human skin fibroblasts. (**a**) Representative Sirius red-stained CCD-966SK cells that received treatments of various concentrations of **DNIC-2** and were incubated for seven days. (**b**) The corresponding collagen contents quantified by extracting the dye and measuring optical density at 540 nm (*n* = 6). The data are presented as mean ± SD. * *p* < 0.05; *** *p* < 0.005; ns, not significant.

Another important physiological function of NO is to modulate the collagen deposition behavior of skin fibroblasts [47,48,49]. Therefore, NO donors hold great promise for various dermatological applications or to act as cosmeceuticals. Herein, to evaluate its capacity to promote collagen deposition, the administration of **DNIC-2** to CCD-966SK cells was performed daily for seven consecutive days. The secreted collagen was detected by staining with Sirius Red that can bind the fibrillar collagens. According to the results in Figure 5a, the fibroblast cultures that received 10 nM or 1 μM **DNIC-2** daily exhibited much denser staining than that of the untreated control. The Sirius Red dye was then extracted followed by quantification using a spectrophotometer. The obtained data further confirmed the increased collagen content in **DNIC-2**-treated groups (*p* < 0.05; Figure 5b), a clear indication of **DNIC-2** in promoting fibroblast collagen deposition. As NO has been identified as a critical mediator for controlling skin collagen accumulation [58], it is no surprise that direct administration of **DNIC-2**, which can release NO for a prolonged duration, can achieve enhanced collagen deposition. Collectively, our results clearly demonstrated that **DNIC-2** could efficiently promote the proliferation, migration, and collagen deposition of skin fibroblast, thus being a promising agent for dermatological applications.

### 2.3. NO-Delivery DNIC-2 Had No Effects on the Pigmentation of Melanocytes

By upregulating the expression level and activity of the key enzyme tyrosinase, NO has been reported as an essential signaling molecule involved in melanogenesis, a process that results in melanin biosynthesis and skin pigmentation [59,60]. To assess the effect of **DNIC-2** on pigment formation, murine B16-F10 melanoma cells that received α-melanocyte-stimulating hormone (α-MSH) induction were used as a model and treated with the developed NO-donor **DNIC-2**. Arbutin (the common whitening agent) was used as a control. After a 48-h incubation, the cell lysate and culture medium were collected for analyzing the intracellular and extracellular melanin content, respectively.

**Figure 6:**
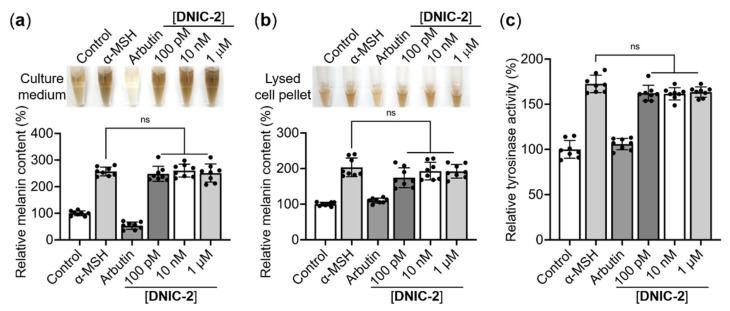
NO-delivery **DNIC-2** does not promote pigmentation of B16-F10 melanoma cells. Representative photos and their corresponding melanin contents of (**a**) the culture media and (**b**) the NaOH-lysed cell pellets of B16-F10 melanoma cells exposed to α-MSH in the presence of **DNIC-2** (*n* = 8). (**c**) Effects of **DNIC-2** on cellular tyrosinase activity (*n* = 8). The data are presented as mean ± SD. ns, not significant.

As revealed in Figure 6a,b, the melanin content in B16-F10 cells and the grown medium was increased after stimulating with α-MSH, while arbutin treatment effectively inhibited the α-MSH-induced melanin synthesis. In all the **DNIC-2**-treated groups, however, changes in the level of melanin content were insignificant. We next analyzed the tyrosinase activity of the cell lysate. As shown in Figure 6c, cell lysate harvested from the **DNIC-2**-treated groups exhibited a similar tyrosinase activity compared to that of α-MSH-stimulated cells. The aforementioned data demonstrated that the developed NO-delivery **DNIC-2** could neither promote nor inhibit the melanogenesis of B16-F10 cells.

### 2.4. Biocompatibility Evaluation of DNIC-2

#### 2.4.1. In Vitro Evaluation of Biocompatibility of DNIC-2 

**Figure 7:**
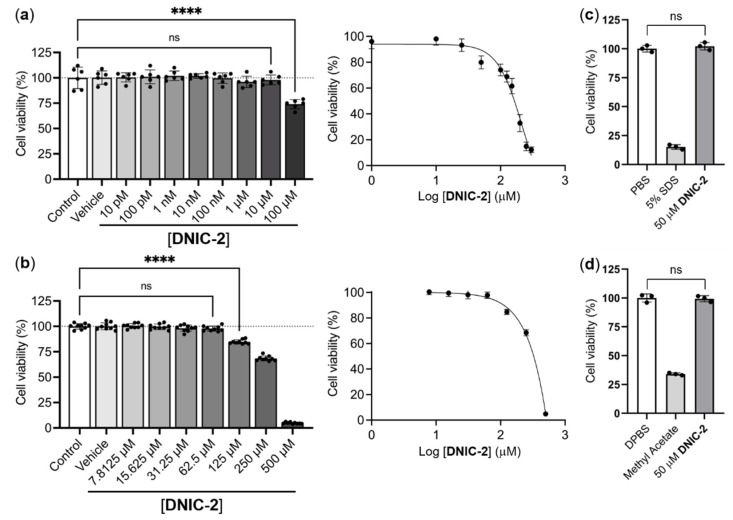
Cell viability assay of the (**a**) mouse skin melanoma (B16-F10) and (**b**) human skin keratinocyte cells (HaCaT) cells, respectively, treated with different concentrations of **DNIC-2** for 24 h **** *p* < 0.001 compared to the group without treatment of **DNIC-2**. (**c**) Cell viability assay of the reconstructed human epidermis (RhE) model treated with PBS, 5% SDS, and 50 μM of **DNIC-2**, respectively. (**d**) Cell viability assay of the reconstructed human cornea-like epithelium model treated with DPBS, methyl acetate, and 50 μM of **DNIC-2**, respectively.

Inspired by the regulatory effect of NO-delivery **DNIC-2** on human fibroblast cells, a biocompatibility evaluation of **DNIC-2** was further performed in the attempt to validate its potential as a novel active ingredient for skincare products. In addition to an IC_50_ value of 92.3 μM toward CCD-966SK human skin fibroblast cells, **DNIC-2** features an IC_50_ value of 222.5 μM and 350.2 μM toward mouse skin melanoma and human skin keratinocyte cells, respectively (Figure 7a,b). Based on the OECD TG 439 Skin Irritation Test and OECD TG 492 Eye Irritation Test, absent toxicity of 50-μM **DNIC-2** toward 3-D cell culture of the reconstructed human epidermis (RhE) and reconstructed human cornea-like epithelium models supports **DNIC-2** as a biocompatible and effective NO-delivery reagent (Figure 7c,d). 

#### 2.4.2. Biocompatibility Evaluation of DNIC-2 in Zebrafish Embryo

**Figure 8:**
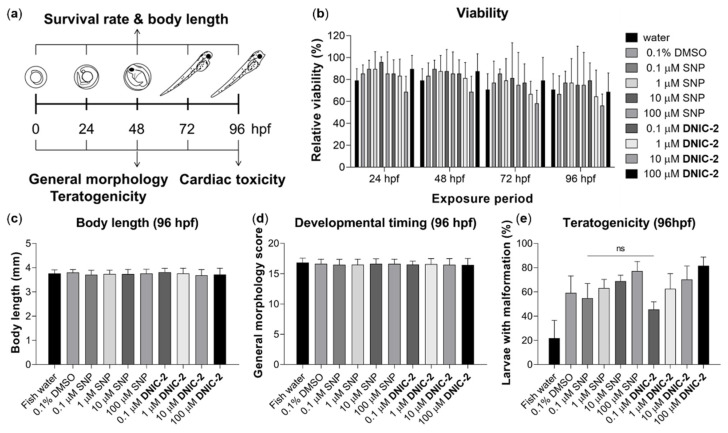
Comparison of developmental toxicity of **DNIC-2** and SNP in embryonic zebrafish. (**a**) Experimental design for ZET assay. (**b**) Viability of zebrafish embryos upon exposure to different concentrations of **DNIC-2** (or SNP) for alternative durations. (**c**) Body length for **DNIC-2**- or SNP-treated zebrafish larvae at 96 hpf. (**d**) General morphology score for **DNIC-2**- or SNP-treated zebrafish larvae at 96 hpf. (**e**) Fraction of larvae with at least one teratogenic effect at 96 hpf. One-way ANOVA with Holm–Šidák test; ns, no significance. Data are expressed as mean + SD (N = 4, *n* = 7–12).

To evaluate the biocompatibility of **DNIC-2**, zebrafish embryos were exposed to different concentrations of **DNIC-2** until 96 h post-fertilization (hpf), whereas a parallel study of FDA-approved sodium nitroprusside (SNP) was also performed (Figure 8a). The survival rate for the control, **DNIC-2**-treated, and SNP-treated groups showed no significant difference (Figure 8b). At 96 hpf, the body length was also comparable among all groups (Figure 8c). 

To further investigate the toxicity of **DNIC-2** on embryonic development, a general morphology scoring system was adopted to evaluate developmental timing and the occurrence of teratogenicity [61]. As shown in Figure 8d, no delay or hastening of embryonic development was observed in **DNIC-2**-treated and SNP-treated groups in comparison with the control group at 96 hpf. For teratogenic assessment, the number of larvae with one or more abnormal anatomical phenotypes at 96 hpf was recorded, and the fraction of larvae with malformation was calculated to infer teratogenicity. The analysis revealed teratogenic tendency seemed to increase in a dose-dependent manner in both **DNIC-2**-treated and SNP-treated groups (Figure 8e). Collectively, these data suggested that the effects of **DNIC-2** on zebrafish developmental toxicity were equivalent to that of SNP.

**Figure 9:**
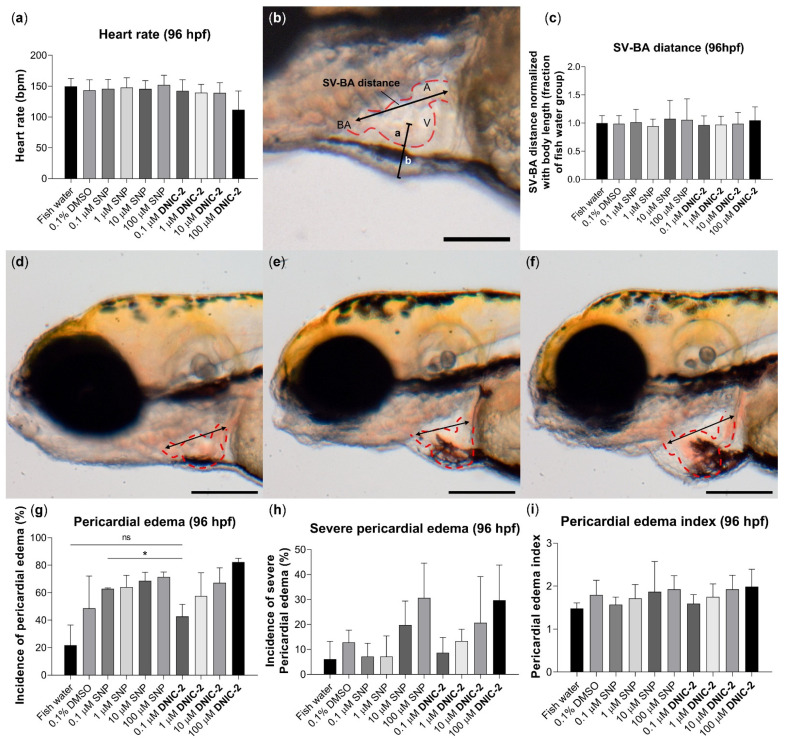
Treatment of **DNIC-2** induced less severe pericardial edema than SNP in zebrafish embryos. (**a**) Heart rate for zebrafish embryos at 96 hpf in the control, **DNIC-2**-treated, and SNP-treated groups. (**b**) Graphical illustration of SV-BA distance and edema index. The measurement of SV-BA distance was indicated with a black arrow, whereas edema index was defined as the ratio of the semi-diameter of the pericardial cavity to that of the ventricle (b/a). Scale bar = 100 μm. (**c**) SV-BA distance normalized with body length. (**d**–**f**) Representative images of cardiac edema in different categories based on SV-BA measurement; the measurement points were indicated by arrows. The hearts were indicated by red dotted lines. Scale bar = 200 μm. (**g**) Fraction of larvae with cardiac edema at 96 hpf. (**h**) Incidence of severe cardiac edema at 96 hpf. (**i**) Pericardial edema index at 96 hpf. One-way ANOVA with Holm–Šidák test; * *p* < 0.05; ns, no significance. Data are expressed as mean + SD (N = 4, *n* = 7–12).

Upon treatment of zebrafish embryos with different concentrations of **DNIC-2** (or SNP), the heart rate, the distance between sinus venosus (SV) and bulbus arteriosus (BA), the incidence of pericardial edema, and the edema index were further investigated in order to assess the potential cardiac toxicity of **DNIC-2** and SNP. At 96 hpf, no significant difference in heart rate and SV-BA distance was observed among all groups (Figure 9a–c), which suggests the absent toxicity of **DNIC-2** and SNP on embryonic heart development. Based on the value of SV-BA distance, the severity of pericardial edema is classified into three categories: normal, mild, and severe groups as shown in Figure 9d–f. As shown in Figure 9g, a treatment of 0.1 μM of **DNIC-2** to zebrafish embryos induced significantly less incidence of pericardial edema than 0.1 μM of SNP treatment. Of interest, **DNIC-2** features a linear trend of dose-dependent induction of cardiac edema, while an exponential trend of dose-dependent induction of cardiac edema was observed when the zebrafish embryo was treated with SNP (Figure 9h). On the other hand, the pericardial edema index indicates similar dose-dependent toxicity of both **DNIC-2** and SNP (Figure 9i). Accordingly, **DNIC-2** demonstrates better biocompatibility than SNP regarding the potential zebrafish embryo cardiac toxicity. 

According to the zebrafish embryotoxicity test (ZET) results described above, pericardial edema induced by NO-delivery **DNIC-2** and SNP, presumably, can be ascribed to the excessive nitrite derived from the oxidation of released nitric oxide [62]. Excessive nitrite was reported to trigger abnormal heart development of zebrafish embryos and to affect the development of the nervous system or muscles [62]. Another potential mechanism for dose-dependent pericardial edema induced by **DNIC-2** (or SNP) in zebrafish embryos is excessive NO-sGC-cGMP signaling, which awaits further verification by exploring the histological and molecular changes of **DNIC-2**-treated embryos with cGMP signaling antagonist [62]. 

## 3. Materials and Methods

Complex [Fe_2_(μ-SCH_2_CH_2_COOH)_2_(NO)_4_] (**DNIC-2**) was synthesized based on published procedures [36]. UV-vis spectra were recorded on a Perkin-Elmer Lambda 365 spectrometer. EPR measurements were performed at X-band using a Bruker EMXmicro-6/1/S/L spectrometer equipped with a Bruker E4119001 super high sensitivity cavity. X-band EPR spectra were obtained with a microwave power of 20.27–20.54 mW, the frequency at 9.41 GHz, conversion time of 66.68 ms, receiver gain of 30, and modulation amplitude of 10.0 G at 100 KHz. Fluorescence intensity was recorded using a microplate reader (SpectraMax iD3, Molecular Devices, San Jose, CA, USA). Fluorescence images were taken using a fluorescence microscope (Olympus, CKX53, Tokyo, Japan). 

**Degradation and NO-release Reactivity of DNIC-2 in MEM.** The aqueous solution for 50 μM of **DNIC-2** was prepared via the addition of 15 μL of a 50-mM stock solution of **DNIC-2** in DMSO to 14.85 mL of MEM. After this solution was incubated at 37 °C under aerobic conditions for 0, 1, 2, 3, 4, 5, 6, 7, 8, 10, 12, 16, and 24 h, UV-vis spectra were measured in order to evaluate the degradation of **DNIC-2** based on the absorbance at 362 nm. Assuming that the degradation of **DNIC-2** follows pseudo-first-order kinetics, the rate for the decrease of A_362_ was calculated to determine the half-life of **DNIC-2** in MEM at 37 °C. Three independent experiments were executed to measure the average half-life for **DNIC-2**. The average half-life for **DNIC-2** in MEM with the presence of 10% FBS at 37 °C was determined in a similar manner.

After the incubation of 25 μM of **DNIC-2** in MEM with or without the presence of 10% FBS under aerobic condition for two days, the equivalent of nitric oxide released from degradation of **DNIC-2** was evaluated using Nitrate/Nitrite Colorimetric Assay Kit (Item No. 780001, Cayman Chemical, Ann Arbor, MI, USA). A general procedure is described below. After 10 μL of the aqueous solution derived from degradation of **DNIC-2** was diluted with 70 μL of kit assay buffer, 10 μL of Nitrate Reductase Cofactors (Item No. 780012) and 10 μL of Nitrate Reductase Enzyme (Item No. 780010) were added before this mixture solution was covered and incubated at room temperature for 2 h. The subsequent addition of 50 μL of Griess Reagent R1 (Item No. 780018) and 50 μL of Griess Reagent R2 (Item No. 780020) followed by incubation at room temperature for 15 min results in the formation of a UV-vis absorption band at 540 nm. The absorbance at 540 nm was then recorded using a microplate reader (SpectraMax iD3, Molecular Devices, San Jose, CA, USA) with a reference wavelength of 800 nm. According to a calibration curve made with 0, 5, 10, 15, 20, 25, 30, and 35 μM of nitrite standard (Item No. 780016)/nitrate standard (Item No. 780014), the equivalent of nitric oxide released from the degradation of **DNIC-2** was further estimated.

The NO-release reactivity of **DNIC-2** was also validated using the fluorescence probe 4-amino-5-methylamino-2′,7′-difluorofluorescein diacetate (DAF-FM, ThermoFisher Scientific, Waltham, MA, USA). In a 96-well black plate, 50 μM of **DNIC-2** and 10 μM of DAF-FM in MEM with the presence of 10% FBS was incubated under aerobic conditions at 37 °C for 0, 0.5, 1, 2, 4, 6, and 8 h. With an excitation wavelength at 495 nm and an emission wavelength at 535 nm, fluorescence intensity was then recorded using a microplate reader (SpectraMax iD3, Molecular Devices, San Jose, CA, USA). Three independent experiments were executed to measure the average fluorescence intensity. The time-dependent change of average fluorescence intensity for 10 μM DAF-FM with or without the treatment of 133 μM of diethylamine NONOate sodium salt hydrate (DEANO, Sigma-Aldrich, St. Louis, MO, USA) in MEM with the presence of 10% FBS at 37 °C was determined in a similar manner. 

**Formation of Protein-bound DNIC upon Incubation of DNIC-2 in MEM.** After the addition of 50 μM of **DNIC-2** into 1 mL of MEM with or without the presence of 10% FBS, the formation of protein-bound DNIC derived from the reaction of **DNIC-2** with serum albumin (2.1 mg/mL) was characterized by EPR spectroscopy. Using DNIC [PPN][(NO)_2_Fe(S_5_)] as a standard, the formation of protein-bound DNIC was quantified based on the calibration curve derived from the EPR spectra of 1, 5, 10, 20, 30, 40, and 50 μM of DNIC [PPN][(NO)_2_Fe(S_5_)] in THF.

**Cell culture.** CCD-966Sk human skin fibroblasts and B16-F10 mouse skin melanoma cells were purchased from Bioresource Collection and Research Center, Food Industry Research and Development Institute (Hsinchu, Taiwan), whereas human skin keratinocyte cell line, HaCaT, was purchased from Elabscience Biotechnology Inc. (Elabscience^®^ EP-CL-0090, Houston, TX, USA). CCD-966Sk cells were cultured in MEM supplemented with 10% FBS, 0.1 mM non-essential amino acids, 1 mM sodium pyruvate, and 1% antibiotic-antimycotic mixture (all from ThermoFisher Scientific, Waltham, MA, USA). B16-F10 cells were cultivated in Dulbecco’s modified Eagle’s medium (phenol red-free; ThermoFisher Scientific, Waltham, MA, USA) supplemented with 10% FBS, 4 mM L-glutamine, and 1% antibiotic-antimycotic mixture. HaCaT cells were cultured in Dulbecco’s modified Eagle’s medium (DMEM; #12100-046, ThermoFisher Scientific, Waltham, MA, USA) containing 5% fetal bovine serum (#10082-147, ThermoFisher Scientific, Waltham, MA, USA) and 1% PSG (Penicillin-Streptomycin-Glutamine (100X) containing 10,000 units of penicillin, 10,000 μg of streptomycin, and 29.2 mg/mL of L-glutamine in a 10 mM citrate buffer (#10378016, ThermoFisher Scientific, Waltham, MA, USA). All cells were grown in a T75 flask at 37°C with 5% CO_2_ in a humidified incubator. The culture medium was refreshed every other day.

**Cellular uptake of DNIC-2 by CCD-966Sk human fibroblast cells.** CCD-966Sk human fibroblast cells were seeded into a 100-mm dish at a density of 1 × 10^6^ cells and incubated at 37 °C overnight. The next day, CCD-966Sk human fibroblast cells were treated with 50 μM of **DNIC-2** and incubated for 0, 0.5, 1, 4, and 8 h, respectively. After removal of the supernatant solution, CCD-966Sk human fibroblast cells were further washed with DPBS before the addition of 1 mL of trypsin/EDTA (0.05%/0.53 mM) and incubation for 5 min at 37 °C. After the obtained cell suspension solution was further centrifuged at 1000× *g* for 5 min, the supernatant was removed before the addition of 100 μL of MEM. This solution was then transferred into the EPR quartz tube and frozen in N_2(l)_ before the EPR measurements. 

**Intracellular release of nitric oxide from DNIC-2 in CCD-966Sk human fibroblast cells.** Intracellular delivery of nitric oxide by **DNIC-2** was validated using the fluorescence probe 4-amino-5-methylamino-2′,7′-difluorofluorescein diacetate (DAF-FM). CCD-966Sk human fibroblast cells were seeded into a 96-well black plate at a density of 2.5 × 10^3^ cells and incubated at 37 °C overnight. The next day, CCD-966Sk human fibroblast cells were first treated with 10 μM of DAF-FM and incubated at 37 °C for 1 h. After removal of the supernatant solution, CCD-966Sk human fibroblast cells were further washed with DPBS. Then, 50 μM of **DNIC-2** was added and incubated for 0, 0.5, 1, 2, 4, and 8 h, respectively, in the dark. The fluorescence intensity of the cells was then recorded using a microplate reader (SpectraMax iD3, Molecular Devices, San Jose, CA, USA) with an excitation wavelength at 495 nm and an emission wavelength at 535 nm. Three independent experiments were executed to measure the time-dependent change of average fluorescence intensity. The time-dependent change of average fluorescence intensity for the CCD-966Sk human fibroblast cells with the treatment of 10 μM of DAF-FM or with the sequential treatment of 10 μM of DAF-FM and 133 μM of DEANO was determined in a similar manner. On the other hand, fluorescence images of the CCD-966Sk human fibroblast cells with the treatment of 10 μM of DAF-FM, with the sequential treatment of 10 μM of DAF-FM and 50 μM of **DNIC-2**, and with the sequential treatment of 10 μM of DAF-FM and 133 μM of DEANO, respectively, were also taken using a fluorescence microscope (Olympus, CKX53, Tokyo, Japan).

**Biological activities of DNIC-2 toward skin fibroblasts**. To evaluate the effects of **DNIC-2** on fibroblast viability, CCD-966SK cells grown in a 96-well plate (2500 cells/well) were treated with various dosages of **DNIC-2**. After incubation for 24 h, cell viability was determined using a CCK-8 assay (IMT Formosa New Materials, Kaohsiung, Taiwan). Alternatively, the grown cells were stained with 0.5 μg/mL DAPI (ThermoFisher Scientific, Waltham, MA, USA) for 20 min, their nuclei were visualized and counted under a fluorescence microscope (Axio Observer 7; Carl Zeiss, Oberkochen, Germany). For each well, six visual fields were selected randomly for nuclei counting. Moreover, live/dead staining was performed using a ViaQuant Viability/Cytotoxicity Kit (GeneCopoeia, Rockville, MD, USA) according to the manufacturer’s instruction.

For the scratch wound healing assay, CCD-966SK cells were seeded into a 24-well plate (4 × 10^4^ cells/well). After a 24-h culture, a P200 tip was utilized to scratch the cellular monolayer to create a cell-free wound in each well. The culture medium was then substituted with a fresh medium with or without **DNIC-2**. The migration of cells into the gap region was photographed under a microscope, and the remaining wound area was determined using ImageJ software [63,64].

To assess the effects of **DNIC-2** on the collagen deposition behavior of skin fibroblasts, CCD-966SK cells were cultured in a 48-well plate (7500 cells/well) for 7 days. The culture medium was replaced with fresh medium with **DNIC-2** every day. To evaluate the collagen content, cells were fixed with 4% paraformaldehyde (Sigma-Aldrich, St. Louis, MO, USA) for 15 min and stained with a Sirius Red/Fast Green Collagen Staining Kit (Chondrex, Woodinville, WA, USA) according to the manufacturer’s protocol [65]. The stained cells were photographed under a microscope before subjecting to dye extraction. The optical density of the extracted dye at 540 nm was determined using a microplate reader to quantify the collagen content of test samples.

**Biological activities of DNIC-2 toward skin melanocytes**. The B16-F10 cells were inoculated into a 12-well plate at a density of 4 × 10^4^ cells per well and incubated for 24 h. Subsequently, under the stimulation of α-MSH (200 nM; Sigma-Aldrich, St. Louis, MO, USA), cells were treated with different concentrations of **DNIC-2** or 2 mM of arbutin (Sigma-Aldrich, St. Louis, MO, USA) for 48 h [66,67,68]. To determine the amount of the melanin secreted by the grown cells, the absorbance of the culture medium at 405 nm was measured using a microplate reader. To quantify the intracellular melanin content, the cells were washed with PBS, trypsinized, pelleted by centrifugation, lysed using 1 N NaOH at 70 °C for 1 h, and analyzed using a microplate reader to measure the optical density at 405 nm [66,67,68].

To determine the activity of tyrosinase, the cells prepared according to the aforementioned methods were lysed with RIPA buffer (20 mM Tris-HCl, 1 mM EGTA, 150 mM NaCl, and 1% Triton X-100) [69] at 4 °C for 20 min followed by centrifugation at 14,000 rpm for 15 min. A bicinchoninic acid assay (G-Biosciences, St. Louis, MO, USA) was used to quantify the total protein of the supernatant. The supernatant that contained 40 µg protein (adjusted to 100 µL with 0.1 mM sodium phosphate buffer; pH 6.8) was mixed with 100 μL of freshly prepared L-DOPA (5 mM; Sigma-Aldrich, St. Louis, MO, USA) and incubated at 37 °C for 1 h [66,70]. Finally, the relative tyrosinase activity of samples was determined by measuring the optical density of the reaction mixture at 475 nm using a microplate reader and normalized to that of the untreated control group.

**Cell viability assay of DNIC-2 toward keratinocyte**. To evaluate the effects of **DNIC-2** on keratinocyte viability, HaCaT cells grown in a 96-well plate (2 × 10^5^ cells/well) were treated with various dosages of **DNIC-2**. After incubation for 24 h, cell viability was determined using an MTT assay (Sigma Aldrich, St. Louis, MO, USA).

**In Vitro Skin Irritation Test by Reconstructed human Epidermis Model.** The validated EPI-200-SIT protocol and the Reconstructed human Epidermis (RhE) model EpiDerm^TM^ provided by MatTek Life Sciences (Ashland, MA, USA) were used for the in vitro skin irritation test (SIT). EpiDerm^TM^ SIT was performed to fulfill the criteria set forth in OECD TG 439 [71]. The EpiDerm^TM^ skin inserts were transferred to 6-well plates containing 0.9 mL EPI-100-NMM-SIT/Assay Medium (MatTek Life Sciences) per well and pre-incubated in the humidified incubator with 5% CO_2_ at 37 °C overnight. Before conducting the SIT test, the assay medium was replaced by the fresh medium and 35 μL of **DNIC-2** (50 μM) was applied topically on top of the EpiDerm^TM^ skin surface for 60 min. Then, DPBS was used to rinse the EpiDerm^TM^ skin surface gently several times, and finally, a sterile cotton swab was gently used to remove excess water and avoid touching the tissue. After that, the EpiDerm^TM^ skin insert was placed in an incubator with 5% CO_2_ at 37 °C for 24 h. Then, the tissue was transferred to a 6-well plate that had been added with a new proprietary medium and incubated again for 18 ± 2 h. After the EpiDerm^TM^ skin inserts were rinsed with PBS twice, 2 mL of MTT reagent (MatTek Life Sciences) was added to the EpiDerm^TM^ skin inserts, which were further incubated in a 5% CO_2_ environment for 3 h before the MTT assay. 2 mL of isopropanol was used to extract formazan. The optical density (OD) of 200 µL of the isopropanol extract was measured at 570 nm using a UV-Vis Spectrophotometer (BioTeK, Winooski, VT, USA). 50 μM of **DNIC-2** was considered to be an irritant to the skin if the tissue viability after exposure was ≤50%. In addition, 5% SDS (Sodium dodecyl sulfate, MatTek Life Sciences, Ashland, MA, USA) was used as a positive control, while DPBS (MatTek Life Sciences, Ashland, MA, USA) was used as a negative control. Three replicates of EpiDerm^TM^ skin inserts were used for each experiment.

**In Vitro Eye Irritation Test by Reconstructed human Cornea-like Epithelium Model.** The validated EPI-200-EIT protocol and the Reconstructed human Cornea-like Epithelium (RhCE) model EpiOcular^TM^ provided by MatTek Life Sciences (Ashland, MA, USA) were used for the in vitro eye irritation test (EIT). EpiOcular^TM^ EIT was performed to fulfill the criteria set forth in OECD TG 492 [72]. The EpiOcular^TM^ cornea inserts were transferred to 6-well plates containing 0.9 mL OCL-200-ASY-Assay Medium (MatTek Life Sciences, Ashland, MA, USA) per well and pre-incubated in the humidified incubator with 5% CO_2_ at 37 °C overnight. Before conducting the SIT test, the assay medium was replaced by the fresh medium and 50 μL of **DNIC-2** (50 μM) was applied topically on top of the EpiOcular^TM^ cornea surface for 30 min. Then, DPBS was used to rinse the EpiOcular^TM^ cornea surface gently several times, and finally, a sterile cotton swab was used to remove excess water and avoid touching the tissue. After that, the EpiOcular^TM^ cornea tissue was soaked in a 5 mL culture medium for 12 min, then transferred to a 12-well plate that had been added with a new proprietary medium, and incubated again for 2 h. Afterward, 2 mL of MTT reagent (MatTek Life Sciences, Ashland, MA 01721) and 2 mL of the medium were added to the EpiDerm^TM^ skin inserts, which were further incubated in a 5% CO_2_ environment for 3 h before the MTT assay. Furthermore, 2 mL of isopropanol was added to the EpiOcular^TM^ cornea inserts and incubated overnight to extract formazan. The optical density (OD) of 200 µL of the isopropanol extract was measured at 570 nm using a UV-Vis Spectrophotometer (BioTeK, Winooski, VT, USA). 50 μM of **DNIC-2** was considered to be an irritant to the eyes if the tissue viability after exposure was ≤60%. In this study, 100% Methyl Acetate (MatTek Life Sciences, Ashland, MA, USA) was used as a positive control, while DPBS (MatTek Life Sciences, Ashland, MA, USA) was used as a negative control. Three replicates of EpiOcular^TM^ cornea inserts were used for each experiment.

**Zebrafish embryotoxicity test (ZET).** All procedures involving animals were approved by the institutional animal experiments committees and performed in compliance with animal welfare laws, guidelines, and policies. (IACUC No. 109022). ZET was carried out following the reported protocol with modifications [61]. Zebrafish embryos were prepared by natural spawning from adult zebrafish and were exposed to **DNIC-2** (or SNP) at different concentrations (between 0.1 and 100 μM) in 2 mL of fresh water from 4 hpf to 96 hpf using 24-well culture plates (4 embryos in 2 mL solution/well). The aqueous solution of **DNIC-2** (or SNP) was renewed every 24 h and the viability of embryos was recorded at the same time. At 48 hpf, the chorion membrane of zebrafish embryos was removed carefully to ensure same exposure condition among all embryos.

To explore the effect of **DNIC-2** (or SNP) on zebrafish embryo development, zebrafish embryos were anesthetized with a drop of 2000-ppm tricaine (Sigma-Aldrich, St. Louis, MO, USA), and then mounted with 2% agarose (Lonza, Basel, Switzerland). Morphological evaluation of zebrafish embryos was performed at 96 hpf using a general morphology scoring system, [61] which comprises the developmental phase up to 96 hpf.

After treatment of different concentrations of **DNIC-2** (or SNP), the heart rate, presence of pericardial edema, and the SV-BA distance in zebrafish embryos were examined to assess drug-induced cardiotoxicity. The evaluation protocol of pericardial edema was modified from previous studies [62,73]. The SV-BA distance normalized to the body length was used to monitor the formation of embryonic heart tubes [74]. The pericardial edema index was defined as the ratio of the semi-diameter of the pericardial cavity to that of the ventricle, which reflects the severity of pericardial edema [62]. Body length, SV-BA distance, and pericardial edema index were measured from a lateral view and processed with Image J software after capturing images using a stereoscopic zoom microscope (SMZ1500; Nikon, Tokyo, Japan) with a microscope camera (DS-Ri2; Nikon, Tokyo, Japan).

## 4. Conclusions

A mechanistic and efficacy study of the cell-penetrating delivery of NO by **DNIC-2** and its regulatory effect on dermato-physiology has led to the following results. **DNIC-2** displays a release of ~4 equiv. of NO with a comparable half-life of 4.2 ± 0.6/3.5 ± 0.3 h in MEM with and without the presence of 10% FBS, respectively. Upon treatment of **DNIC-2** to CCD-966SK human skin fibroblast cells, cellular uptake of **DNIC-2** followed by its intracellular transformation into protein-bound DNICs rationalizes the steady and effective release of NO with an intracellular half-life of 1.8 ± 0.2 h, which overwhelms the NO donor diethylamine NONOate (DEANO). The treatment of skin fibroblasts with low-dose **DNIC-2** resulted in enhanced cell proliferation, migration, and collagen deposition, thus holding substantial potential for dermatological applications. On the other hand, the application of **DNIC-2** to melanoma cells did not lead to significant alternation in melanogenesis. Based on the biocompatibility evaluation using a 2-D culture of fibroblast/melanocyte/keratinocyte cells, the 3-D culture of reconstructed human epidermis and human cornea-like epithelium models, and zebrafish embryo toxicity assay, **DNIC-2** holds the potential for further development as a novel active ingredient for skincare products.

## Supplementary Materials

The following are available online at https://www.mdpi.com/article/10.3390/ijms221810101/s1.
