# Supplementary material for: Cell-Penetrating Delivery of Nitric Oxide by Biocompatible Dinitrosyl Iron Complex and Its Dermato-Physiological Implications
Source: Int J Mol Sci. 2021 Sep 18;22(18):10101. doi: 10.3390/ijms221810101 (PMC8469893; doi:10.3390/ijms221810101)
Supplement: Supplementary file 1 [file ijms-22-10101-s001.zip › ijms-1371067-supplementary.pdf]

Supporting Information

Cell-Penetrating Delivery of Nitric Oxide by Biocompatible Dinitrosyl Iron Complex and Its Dermato-physiological Implications

Yu-Chieh Chen [1,#], Yi-Hong Chen [2,#], Han Chiu [1], Yi-Hsuan Ko [1], Ruei-Ting Wang [3], Wei-Ping Wang [3], Yung-Jen Chuang [1], Chieh-Cheng Huang [2,*], and Tsai-Te Lu [2,*]

Affiliations:

[1]Department of Medical Science & Institute of Bioinformatics and Structural Biology, National Tsing Hua University, Hsinchu, 30013, Taiwan.

[2]Institute of Biomedical Engineering, National Tsing Hua University, Hsinchu, 30013, Taiwan.

[3]CHLITINA Research and Development Center, CHLITINA Holding Ltd, Taipei, 10073, Taiwan.

[#]These authors contributed equally: Yu-Chieh Chen, Yi-Hong Chen.

[*]Corresponding author:

Chieh-Cheng Huang, Ph.D., Institute of Biomedical Engineering, National Tsing Hua University, Hsinchu, Taiwan. E-mail: chiehcheng@mx.nthu.edu.tw

Tsai-Te Lu, Ph.D., Institute of Biomedical Engineering, National Tsing Hua University, Hsinchu, Taiwan. E-mail: ttlu@mx.nthu.edu.tw.

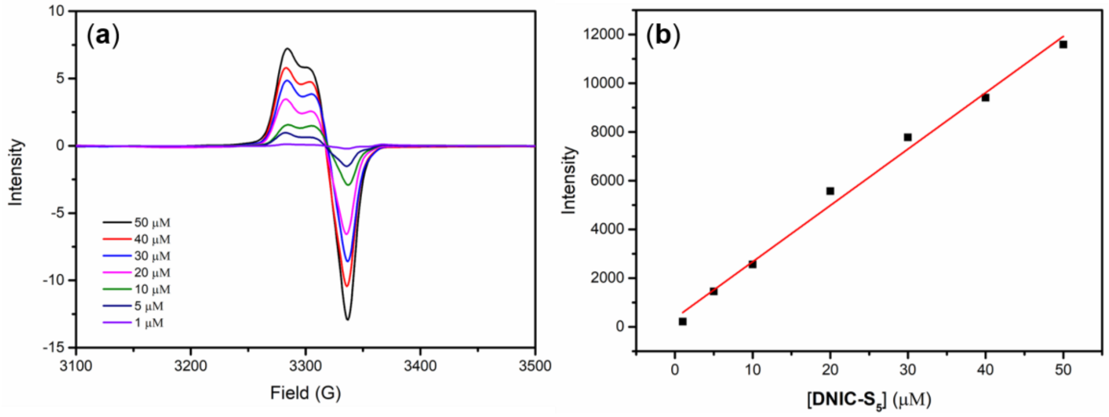

Figure S1. (**a**) EPR spectra for different concentration of DNIC [PPN][$(NO)_2Fe(S_5)$] in THF. (**b**) Calibration curve [PPN][$(NO)_2Fe(S_5)$] in THF.

Table S1. Chemical Composition of Minimum Essential Media (MEM).

Component	MEM
pH	7.4
Cl^{-} (mM)	126.6
HCO_3^{-}/H_2CO_3 (mM)	26.2
$HPO_4^{2-}/H_2PO_4^{-}$(mM)	1.0
SO_4^{2-} (mM)	0.8
Na^{+} (mM)	144.4
K^{+} (mM)	5.3
Ca^{2+} (mM)	1.8
Mg^{2+} (mM)	0.8
Glucose (mM)	5.6
Amino acids (mM)	3.4
Choline chloride (μM)	7.1
D-Calcium pantothenate (μM)	2.1
Folic Acid (μM)	2.3
Niacinamide (μM)	8.2
Pyridoxal hydrochloride (μM)	4.9
Riboflavin (μM)	0.3
Thiamine hydrochloride (μM)	3.0
i-Inositol (μM)	11.1

Table S2. Major Chemical Composition of Fetal Bovine Serum (FBS).

Component	100% FBS	10% FBS
pH	6.5 - 8.5	6.5 - 8.5
Cl^{-} (mM)	99	9.9
Phosphorus (mM)	3.07	0.31
Na^{+} (mM)	136	13.6
K^{+} (mM)	>10	>1
Ca^{2+} (mM)	3.38	0.34
Iron (μM)	29.7	2.97
Glucose (mM)	5.5	0.55
Protein (total, mg/mL)	35	3.5
Albumin (mg/mL)	21	2.1
Urea (mM)	2.33	0.23
Bilirubin (mg/L)	3	0.3
Cholesterol (mg/L)	300	30
Creatinine (mg/L)	25	2.5
Globulin (mg/L)	14	1.4
Hemoglobin (mg/L)	133	13.3
IgG (μg/mL)	89	8.9
